# Early Intraprosthetic Dislocation of Total Hip Arthroplasty with Double Mobility Implant: Case Report

**DOI:** 10.1055/s-0041-1726068

**Published:** 2022-09-26

**Authors:** Thiago Lopes Lima, Alexandre de Bustamante Pallottino, José Sérgio Franco, Sávio Manhães Chami, Breno Jorge Scorza, Brunno Benedetti de Morais

**Affiliations:** 1 Casa de Saúde São José, Rio de Janeiro, RJ, Brasil; 2 Serviço de Ortopedia e Traumatologia Prof. Nova Monteiro, Hospital Municipal Miguel Couto, Rio de Janeiro, RJ, Brasil; 3 Departamento de Ortopedia e Traumatologia, Faculdade de Medicina, Universidade Federal do Rio de Janeiro, Rio de Janeiro, RJ, Brasil; 4 Serviço de Ortopedia, Casa de Saúde São José, Rio de Janeiro, RJ, Brasil

**Keywords:** arthroplasty, replacement, hip, hip, hip dislocation, periprosthetic fractures

## Abstract

Total hip arthroplasty (THA) is a successful surgery in the treatment of hip pain, but there are potential complications, of which dislocation is one of the most common. Dislocation management is a challenging problem that requires a multimodal approach, and the use of dual mobility implants is an option. We present a patient with a history of femoral neck fracture who underwent THA with a double mobility implant. On the 18 ^th^ postoperative day, after a fall to the ground, she developed prosthesis dislocation and had a complication after closed reduction, a subsequent intraprosthetic dislocation. After a radiographic diagnosis, the patient presented mechanical signs of hip flexion caused by a disassociated double mobility implant. The revision surgery was indicated, but the patient chose not to perform the necessary surgical procedure. A careful postoperative study of the radiographs revealed an eccentric femoral head and evidence of disassociated implantation in the surrounding soft tissues. Radiographs after closed reduction of intraprosthetic dislocations should be examined thoroughly.

## Introduction

Instability is one of the most feared complications of hip arthroplasty, corresponding from between 0.2 and 7% of primary arthroplasties to 25% in review cases. 1

In the 1970s, Giles Bousquet, aiming to reduce this index, developed the concept of double mobility. This consists of two joints, a larger one between the polyethylene liner and the metal acetabular cupula, and the other, smaller, between the femoral head and the polyethylene liner, increasing the radius between the head and the neck and the distance to impact between the neck and the acetabular edge, reducing dislocation rates in this type of prosthesis. 2

In this implant, early intraprosthetic dislocation (dissociation between the femoral head and polyethylene) is extremely rare, with few cases described. The causes of early intraprosthetic dislocation are inadequate assembly of components and dislocation or forced reduction. Other causes are the extrinsic blockage of polyethylene coating or polyethylene wear (debris would compromise the coupling of the two bearings), which occurs years after the procedure, not being common in early dislocation. 3

Radiographic recognition of intraprosthetic dislocation after reduction may be difficult, because the head of the femoral component may be located within the metallic acetabular cup, while the polyethylene component is displaced. Radiographic signs of an intraprosthetic dislocation include eccentricity of the femoral head inside the metallic acetabulum and the presence of a "bubble sign" on postreduction radiography, representing polyethylene. 4 Immediate recognition is essential for planning open reduction and exchange of polyethylene liner.

## Case Report

**Fig. 1:**
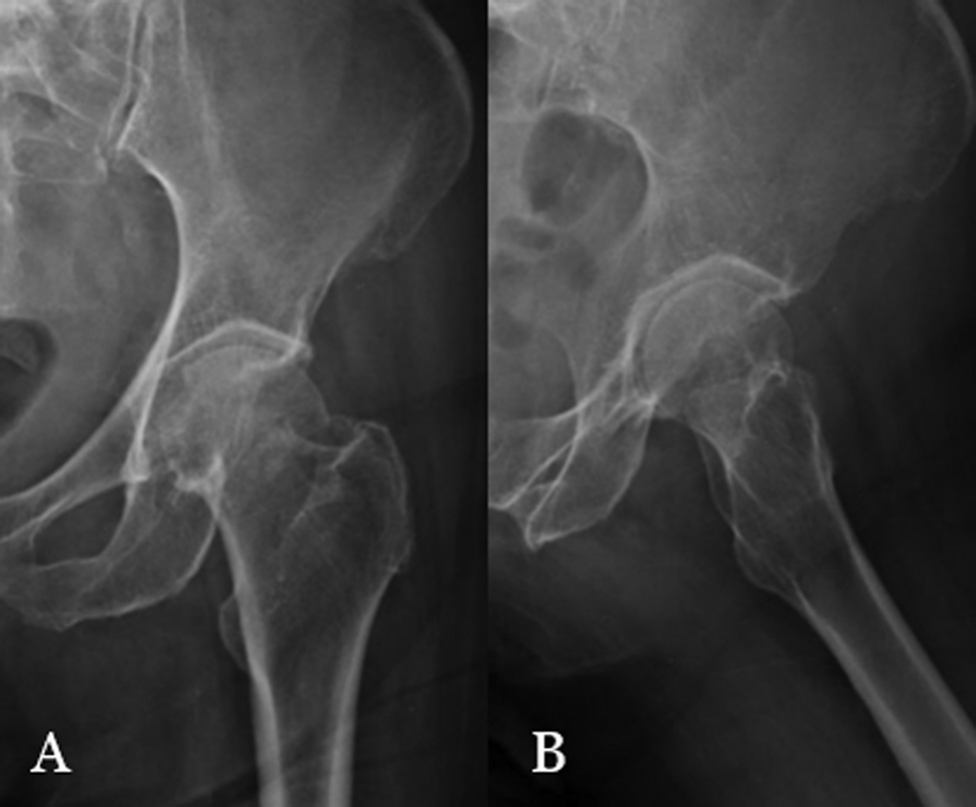
Fracture of the neck of the left femur impacted in valgus, in AP (A) and profile (B).

Female patient, 85 years old, with a history of fall to the ground. She was treated in the emergency room with hip pain, shortening and external rotation in the left lower limb. The radiographic examination revealed the diagnosis of fracture of the neck of the left femur, AO 31B1.1 ( Fig. 1a-b ).

The patient underwent total arthroplasty of the left hip with double mobility implant (Medacta). A 48/28 double mobility polyethylene liner, size 1 uncemented femoral stem, a 28 mm metallic femoral head, and a 48 mm noncemented acetabular dome were used. Posterolateral access was performed with capsular repair and short rotators.

The choice of the double mobility prosthesis occurred due to risk factors for instability: age > 75 years, flexibility, hypermobility, active life, female gender. 5

**Fig. 2:**
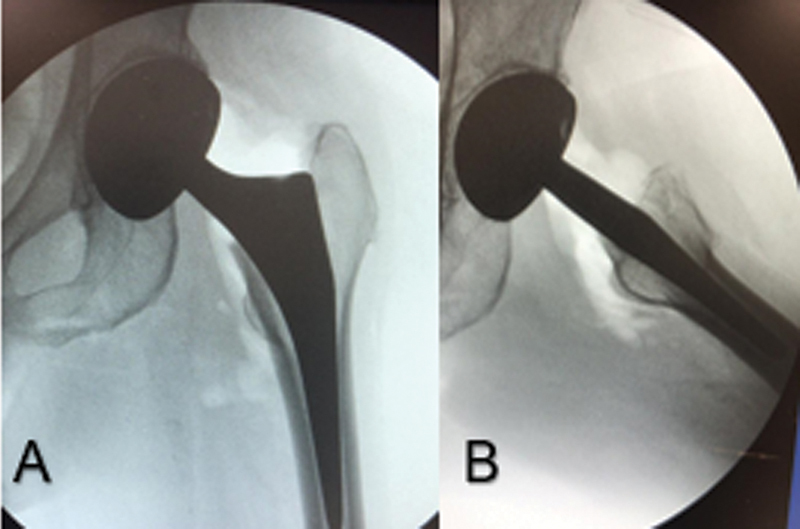
Radiographic control in the immediate postoperative period showing reduction of components and concentricity of the metallic head (A) in AP and profile (B).

The polyethylene liner was properly coupled to the metal head with specific material and tested before being deployed. Stability tests and intraoperative control radiographs were performed, confirming good implant positioning and hip stability ( Fig. 2a-b ).

**Fig. 3:**
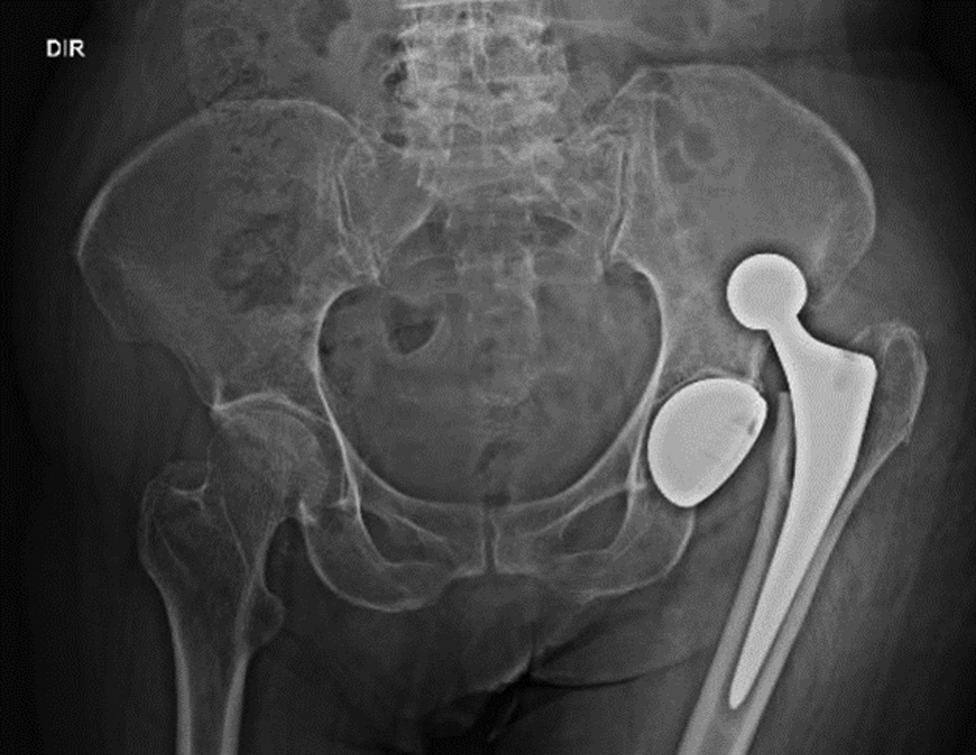
Panoramic radiography of the basin showing posterosuperior dislocation of the left hip prosthesis.

On the 18 ^th^ postoperative day, after a fall to the ground, the patient was treated in the emergency room, complaining of pain in her left hip. Radiographs of the hip showed upper posterior dislocation of the prosthesis ( Fig. 3 ).

**Fig. 4:**
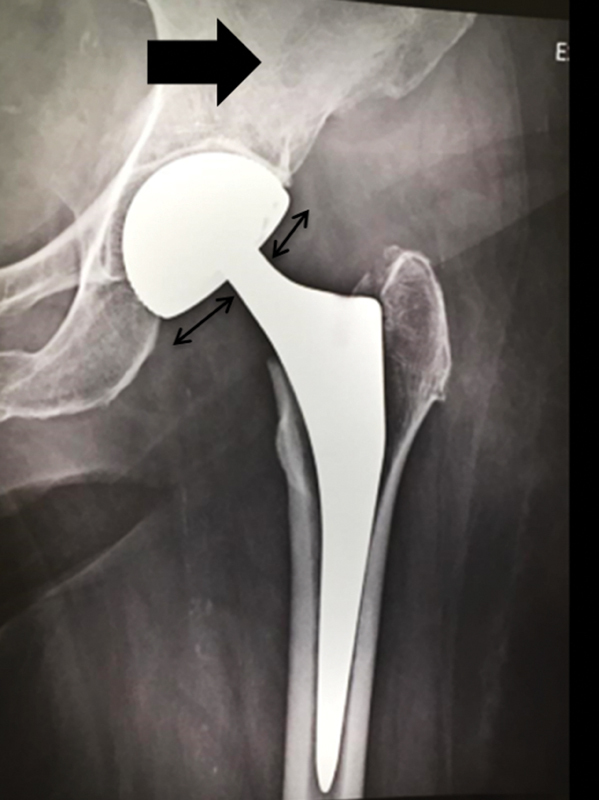
X-ray 7 days after reduction. The larger arrow shows the "bubble sign", the polyethylene displaced in the soft parts. The smaller arrows show the eccentricity of the metal head in the acetabular dome.

**Fig. 5:**
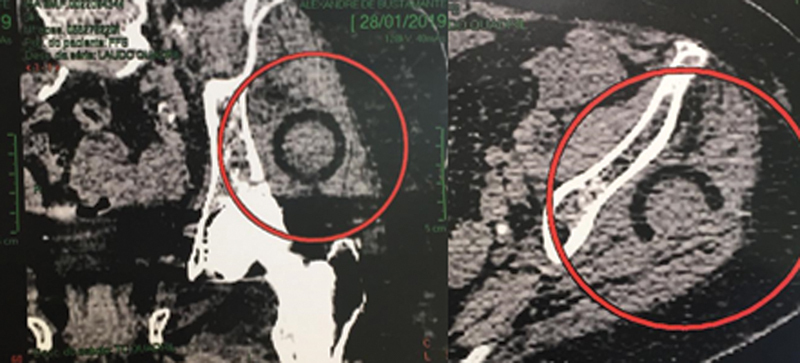
(A) Coronal section of computed tomography. The red circle shows polyethylene component in soft tissues, (B) In the axial cut, a red circle evidencing polyethylene.

The closed reduction was performed with sedation (propofol), using the reduction maneuver of Allis apud Waddell et al. 6 and radiographic control. She returned to the outpatient clinic on the 7 ^th^ day after reduction in good general condition, without functional limitation, complaining only of crackling in the posterior region of the left hip. Radiographs after reduction show congruence between the femoral head and the acetabular dome, but femoral head eccentricity is observed in the acetabular metallic dome ( Fig. 4 ). A superolateral circular opacity (bubble sign) is also observed. Computed tomography (CT) reveals a polyethylene component in the posterosuperior aspect of the hip ( Fig. 5 ).

The patient underwent outpatient follow-up. She can perform all daily activities without functional limitation. The only complaint was polyethylene crackling which improved with ∼ 60 days. She did not present other episodes of dislocation. Due to the absence of symptoms and high surgical risk (chronic renal failure and severe heart disease), both the patient and the responsible physician chose not to perform revision surgery.

## Discussion

In our case, the patient presented complications after a single attempt at closed reduction, in which the maneuver strength resulted in intraprosthetic displacement of the polyethylene. This complication occurs when the polyethylene coating fits into the edge of the acetabular component, and the subsequent traction of the limb results in the dissociation of the metal head from the polyethylene coating, similar to a "bottle opener" effect, and polyethylene can migrate out of the acetabulum.

Intraprosthetic dislocation can be a diagnostic challenge, especially in postreduction radiographs, because the femoral head can give the false impression of being reduced in the dome.

When evaluating a dislocation after hip arthroplasty, it is necessary to differentiate a conventional prosthesis from a double mobility implant. The presence of crackling or noise associated with the request of the motion arc is a sign of direct contact between the head and the acetabular dome. The x-ray should be made with close attention to the eccentricity of the head in the acetabular component. The possible presence of an opaque radio halo in the periarticular region ("bubble sign"), which may represent dislocated polyethylene, should also be observed.

The reduction in these patients should be performed with general anesthesia or subarachnoid block, in order to facilitate it. The maneuver should be performed carefully and without axial traction to avoid or attenuate the "bottle opener" effect. Instead of applying direct axial traction, internal rotation should be coupled to axial traction, allowing the polyethylene coating to move away from the acetabulum, avoiding the collision of the acetabular component. Fluoroscopic imaging should be used to guide the reduction maneuver. Postreduction radiographs should be carefully evaluated for femoral head eccentricity and the presence of a bubble sign. Intraprosthetic dislocation requires surgical intervention, and anesthesia should be used for open reduction and eventual component replacement. Computed tomography may be requested in case of doubt. The suggestion would be to place a metallic marker on the polyethylene that would facilitate its detection on radiographs.

With the popularization of dual mobility prostheses in our country, training and information of orthopedists are needed in the emergency room for this possible complication that is easy to be neglected. The examining physician should question and know how to identify the implant model and evaluate the signs of this complication: femoral head eccentricity in the acetabular dome, "soft tissue bubble sign", crackling or deformity on joint palpation after reduction.

## References

[1] Patel P D Potts A Froimson M I The dislocating hip arthroplasty: prevention and treatment J Arthroplasty 2007 22(04, Suppl 1):86 90 17570285 10.1016/j.arth.2006.12.111

[2] McArthur B A Nam D Cross M B Westrich G H Sculco T P Dual-mobility acetabular components in total hip arthroplasty Am J Orthop 2013 42 10 473 478 24278908

[3] Philippot R Adam P Farizon F Fessy M H Bousquet G [Survival of cementless dual mobility sockets: ten-year follow-up] Rev Chir Orthop Repar Appar Mot 2006 92 04 326 331 10.1016/s0035-1040(06)75762-2 16948459

[4] De Martino I Triantafyllopoulos G K Sculco P K Sculco T P Dual mobility cups in total hip arthroplasty World J Orthop 2014 5 03 180 187 25035820 10.5312/wjo.v5.i3.180 PMC4095010

[5] Kaiser D Kamath A F Zingg P Dora C Double mobility cup total hip arthroplasty in patients at high risk for dislocation: a single-center analysis Arch Orthop Trauma Surg 2015 135 12 1755 1762 26419896 10.1007/s00402-015-2316-5

[6] Waddell B S Mohamed S Glomset J T Meyer M S A Detailed Review of Hip Reduction Maneuvers: A Focus on Physician Safety and Introduction of the Waddell Technique Orthop Rev (Pavia) 2016 8 01 6253 27114811 10.4081/or.2016.6253 PMC4821229

