# Supplementary material for: Early Intraprosthetic Dislocation of Total Hip Arthroplasty with Double Mobility Implant: Case Report
Source: Rev Bras Ortop (Sao Paulo). 2022 Sep 26;59(3):e475–8. doi: 10.1055/s-0041-1726068 (PMC11193582; doi:10.1055/s-0041-1726068)
Supplement: Related texts

# Research article: Luxação intraprotética precoce de artroplastia total do quadril com implante de dupla mobilidade: Relato de caso

**Keywords:** artroplastia de quadril, fraturas periprotéticas, luxação do quadril, quadril

## Abstract

A artroplastia total do quadril (ATQ) é uma cirurgia bem-sucedida no tratamento da dor no quadril, mas existem complicações potenciais, das quais a luxação é uma das mais comuns. O gerenciamento das luxações é um problema desafiador que requer uma abordagem multimodal, e o uso de implantes de mobilidade dupla é uma opção. Apresentamos uma paciente com história de fratura do colo do fêmur que foi submetida a ATQ com um implante de dupla mobilidade. No 18° dia pós-operatório, após queda ao solo, a paciente evoluiu com luxação da prótese e teve uma complicação após redução fechada, uma luxação intraprotética subsequente. Após um diagnóstico radiográfico, a paciente apresentou sinais mecânicos na flexão do quadril causados por um implante de mobilidade dupla desassociado. A cirurgia de revisão foi indicada, mas a paciente optou por não realizar o procedimento cirúrgico necessário. O estudo pós-operatório cuidadoso das radiografias revelou uma cabeça femoral excêntrica e evidências do implante desassociado nos tecidos moles circundantes. As radiografias após a redução fechada das luxações intraprotéticas devem ser examinadas minunciosamente.

## Introdução

A instabilidade é uma das mais temidas complicações da artroplastia do quadril, correspondendo a entre 0,2 e 7% das artroplastias primarias e a 25% nos casos de revisão. 1

Na década de 1970, Giles Bousquet, visando reduzir este índice, desenvolveu o conceito de dupla mobilidade. Este consiste em duas articulações, uma maior entre o liner de polietileno e a cúpula acetabular metálica e outra menor entre a cabeça femoral e o liner de polietileno, aumentando o raio entre a cabeça e o colo e a distância para impacto entre o colo e a borda acetabular, reduzindo as taxas de luxação neste tipo de prótese. 2

Neste implante, a luxação intraprotética (dissociação entre a cabeça femoral e o polietileno) precoce é extremamente rara, com poucos casos descritos. As causas de luxação intraprotética precoce são a montagem inadequada dos componentes e a luxação ou redução forçada. Outras causas são o bloqueio extrínseco do revestimento de polietileno ou desgaste do polietileno (debris comprometeriam o acoplamento dos dois rolamentos) que ocorre anos após o procedimento, não sendo comum na luxação precoce. 3

O reconhecimento radiográfico da luxação intraprotética após a redução pode ser difícil, porque a cabeça do componente femoral pode estar localizada dentro da taça acetabular metálica, enquanto o componente de polietileno é deslocado. Sinais radiográficos de uma luxação intraprotética incluem a excentricidade da cabeça femoral no interior do acetábulo metálico e a presença de um “sinal de bolha” na radiografia de pós-redução, que representa o polietileno. 4 O reconhecimento imediato é essencial para o planejamento da redução aberta e troca do liner de polietileno.

## Relato de Caso

Paciente do sexo feminino, 85 anos, com história de queda ao solo, atendida na emergência com dor no quadril, encurtamento e rotação externa no membro inferior esquerdo. O exame radiográfico revelou o diagnóstico de fratura do colo do fémur esquerdo, AO 31B1.1 ( Fig. 1a-b ).

**Fig. 1:** Fratura do colo do fêmur esquerdo impactada em valgo, em AP (A) e perfil (B).

A paciente foi submetida a artroplastia total do quadril (ATQ) esquerdo com implante de dupla mobilidade (Medacta). Utilizou-se liner de polietileno de dupla mobilidade 48/28, haste femoral não cimentada tamanho 1, cabeça femoral metálica de 28 mm, é cúpula acetabular de 48 mm não cimentada. Foi realizado acesso posterolateral com reparo capsular e dos rotadores curtos.

A escolha da prótese de dupla mobilidade ocorreu por conta dos fatores de risco para instabilidade: idade > 75 anos, flexibilidade, hipermobilidade, vida ativa, sexo feminino. 5

O liner de polietileno foi acoplado apropriadamente na cabeça metálica com material específico e testado antes de ser implantado. Testes de estabilidade e radiografias de controle intraoperatórios foram realizados, confirmando o bom posicionamento dos implantes e a estabilidade do quadril ( Fig. 2a-b ).

**Fig. 2:** Controle radiográfico no pós-operatório imediato evidenciando redução dos componentes e concentricidade da cabeça metálica (A) em AP e perfil (B).

No 18° dia pós-operatório, a paciente, após queda ao solo, foi atendida na emergência com queixa de dor no quadril esquerdo. Radiografias do quadril evidenciaram luxação posterosuperior da prótese ( Fig. 3 ).

**Fig. 3:** Radiografia panorâmica da bacia evidenciando luxação póstero superior da prótese do quadril esquerdo.

A redução fechada foi realizada com sedação (propofol), utilizando a manobra de redução de Allis apud Waddell et al. 6 e controle radiográfico. A paciente retornou ao ambulatório no 7° dia após a redução em bom estado geral, sem limitação funcional, queixando-se apenas de crepitação na região posterior do quadril esquerdo. As radiografias pós-redução evidenciaram congruência entre a cabeça femoral e a cúpula acetabular, porém observa-se excentricidade da cabeça femoral na cúpula metálica acetabular ( Fig. 4 ). Observa-se também uma opacidade circular (sinal da bolha) superolateralmente. Uma tomografia computadorizada (TC) revela o componente de polietileno no aspecto posterosuperior do quadril ( Fig. 5 ).

**Fig. 4:** Raio X 7 dias após a redução. A seta maior mostra o “sinal da bolha”, o polietileno deslocado nas partes moles. As setas menores evidenciam a excentricidade da cabeça metálica na cúpula acetabular.

**Fig. 5:** (A) Corte coronal da tomografia computadorizada. Círculo vermelho evidencia componente de polietileno nos tecidos moles, (B) No corte axial, círculo vermelho evidenciando polietileno.

Paciente em acompanhamento ambulatorial. Consegue realizar todas as atividades do cotidiano sem limitação funcional. A única queixa era o crepitar do polietileno, que melhorou em ∼ 60 dias. A paciente não apresentou outros episódios de luxação. Por conta da ausência de sintomas e risco cirúrgico elevado (insuficiência renal crônica e cardiopatia grave), tanto a paciente quanto o clínico responsável optaram por não realizar a cirurgia de revisão.

## Discussão

No nosso caso, a paciente apresentou complicação após uma única tentativa de redução fechada, na qual a força da manobra resultou em deslocamento intraprotético do polietileno. Esta complicação ocorre quando o revestimento de polietileno encaixa na borda do componente acetabular, e a subsequente tração do membro resulta na dissociação da cabeça metálica do revestimento de polietileno, semelhante a um efeito “abridor de garrafas”, podendo o polietileno migrar para fora do acetábulo.

A luxação intraprotética pode ser um desafio diagnóstico, principalmente nas radiografias pós-redução, porque a cabeça femoral pode dar a falsa impressão de estar reduzida na cúpula.

Ao avaliar uma luxação após artroplastia de quadril, é necessário diferenciar uma prótese convencional de um implante de dupla mobilidade. A presença de crepitação ou ruído associada à solicitação do arco de movimento é sinal de contato direto entre a cabeça e a cúpula acetabular. Ao Raio X, deve-se atentar quanto à excentricidade da cabeça no componente acetabular. Deve-se observar também a possível presença de um halo radio opaco na região periarticular (“sinal da bolha”), que pode representar o polietileno luxado.

A redução desses pacientes deve ser realizada com anestesia geral ou bloqueio subaracnóideo, visando facilitar a redução. A manobra deve ser feita de forma cuidadosa e sem tração axial, para evitar ou atenuar o efeito “abridor de garrafa”. Em vez de aplicar tração axial direta, a rotação interna deve ser acoplada à tração axial, permitindo que o revestimento de polietileno se afaste do acetábulo, evitando a colisão do componente acetabular. A imagem fluoroscópica deve ser usada para guiar a manobra de redução. As radiografias de pós-redução devem ser cuidadosamente avaliadas quanto à excentricidade da cabeça femoral e a presença de um sinal de bolha. A luxação intraprotética requer intervenção cirúrgica, devendo ser aproveitada a anestesia para redução aberta e eventual troca de componentes. A TC pode ser solicitada em caso de dúvidas. A sugestão seria colocar um marcador metálico no polietileno, o qual facilitaria sua detecção nas radiografias.

Com a popularização das próteses de dupla mobilidade no nosso meio, se faz necessário treinamento e informação dos ortopedistas no pronto atendimento para essa possível complicação fácil de ser negligenciada. O médico examinador deve questionar e saber identificar o modelo de implante e avaliar os sinais dessa complicação: excentricidade da cabeça femoral na cúpula acetabular, “sinal da bolha em partes moles”, crepitação ou deformidade à palpação da articulação após a redução.
